# A Further Look at Therapeutic Interventions for Suicide Attempts and Self-Harm in Adolescents: An Updated Systematic Review of Randomized Controlled Trials

**DOI:** 10.3389/fpsyt.2018.00583

**Published:** 2018-11-23

**Authors:** Udita Iyengar, Natasha Snowden, Joan R. Asarnow, Paul Moran, Troy Tranah, Dennis Ougrin

**Affiliations:** ^1^Department of Child and Adolescent Psychiatry, Institute of Psychiatry, Psychology, and Neuroscience (IoPPN), King's College London, London, United Kingdom; ^2^Semel Institute of Neuroscience and Behavior, David Geffen School of Medicine, University of California, Los Angeles, Los Angeles, CA, United States; ^3^National Institute for Health Research Bristol Biomedical Research Centre, University Hospitals Bristol NHS Foundation Trust and University of Bristol, Bristol, United Kingdom; ^4^Department of Population Health Sciences, Centre for Academic Mental Health, Bristol Medical School, University of Bristol, Bristol, United Kingdom; ^5^Institute of Psychiatry, Psychology, and Neuroscience (IoPPN), London, United Kingdom; ^6^South London and Maudsley National Health Service (NHS) Foundation Trust, London, United Kingdom

**Keywords:** suicide, self-harm, NSSI, depression, suicidal ideation, adolescent, RCT

## Abstract

**Background:** Suicide attempts (SA) and other types of self-harm (SH) are strong predictors of death by suicide in adolescents, emphasizing the need to investigate therapeutic interventions in reduction of these and other symptoms. We conducted an updated systematic review of randomized controlled trials (RCTs) from our previous study reporting therapeutic interventions that were effective in reducing SH including SA, while additionally exploring reduction of suicidal ideation (SI) and depressive symptoms (DS).

**Method:** A systematic literature search was conducted across OVID Medline, psycINFO, PubMed, EMBASE, and Cochrane Library from the first available article to October 22nd, 2017, with a primary focus on RCTs evaluating therapeutic interventions in the reduction of self-harm. Search terms included *self-injurious behavior; self-mutilation; suicide, attempted; suicide; drug overdose*.

**Results:** Our search identified 1,348 articles, of which 743 eligible for review, yielding a total of 21 studies which met predetermined inclusion criteria. Eighteen unique therapeutic interventions were identified among all studies, stratified by individual-driven, socially driven, and mixed interventions, of which 5 studies found a significant effect for primary outcomes of self-harm and suicide attempts (31.3%), and 5 studies found a significant effect for secondary outcomes of suicidal ideation and depressive symptoms (29.4%) for therapeutic intervention vs. treatment as usual. Collapsing across different variations of Cognitive Behavior Therapy (CBT), and classifying Dialectical Behavior Therapy for Adolescents (DBT-A) as a type of CBT, CBT is the only intervention with replicated positive impact on reducing self-harm in adolescents.

**Conclusion:** While the majority of studies were not able to determine efficacy of therapeutic interventions for both primary and secondary outcomes, our systematic review suggests that individual self-driven and socially-driven processes appeared to show the greatest promise for reducing suicide attempts, with benefits of combined self-driven and systems-driven approaches for reducing overall self-harm. Further RCTs of all intervention categories are needed to address the clinical and etiological heterogeneity of suicidal behavior in adolescents, specifically suicidal ideation and depressive symptoms.

## Introduction

Suicide is a major global and public health concern (1). It is the second leading cause of death in people age 15–24 years (2) and there is a pressing need to identify effective interventions to reduce the risk of suicide. Non-fatal suicide attempts (SA) can be defined as self-directed injuries with implicit or explicit intent to kill oneself, while non-suicidal self-injury (NSSI) is direct destruction of one's body without intention to die (3). For the latter, it is useful to consider the definition of non-suicidal self-injury found in the DMS-5, which states that the preoccupied individual partakes in premeditated, self-directed damage to themselves in order to relieve negative experiences and does not exhibit suicidal intent through this behavior (4). Both suicide attempts (SA) and the broader category of “self-harm” (SH, which includes non-suicidal self-injury) are among the strongest predictors of death by suicide (5–7), and have therefore appropriately been the focus of therapeutic interventions for adolescents to decrease risk of suicide.

There has been significant progress in detection (8), identifying subtypes, understanding the long-term outcomes (9), and understanding help-seeking in adolescents with SH (10). There has also been recent progress regarding the treatment of self-harm in adolescents. We conducted the first meta-analysis of randomized controlled trials (RCTs) to specifically evaluate therapeutic interventions (TIs) in reducing SH in adolescents (11). A significant effect was found for tested interventions reducing SH compared to treatment as usual (TAU). While results evaluating the effects of therapeutic interventions on NSSI were generally consistent with those for overall self-harm, the effect size was weaker and escaped statistical significance. In contrast, there was little to no evidence of benefits of tested interventions in reducing suicide attempts. Our findings highlighted both the beneficial effects of therapeutic interventions for self-harm as a global category, the challenges of reducing the risk of future suicide attempts and the need for rigorous and replicable studies.

In addition to self-harm and suicide attempts, however, depression in adolescence is another key contributor to suicidal behavior (12, 13). A recent National Confidential Inquiry into Suicide and Homicide by People with Mental Illness (NCISH) report of suicide deaths in England and Wales between 2014 and 2015 indicated that out of 285 suicide deaths that occurred in youths aged 10–20, 52% had a history of SH, while 58% expressed thoughts of suicide or hopelessness (14). Depressive symptoms themselves have been found to be significant and independent contributors to elevated levels of deliberate self-harm in young people (15–18). Therefore, it would appear that the reduction of depressive symptoms and suicidal ideation (19) may be an important mechanism underpinning the effectiveness of certain treatments for suicide prevention.

We therefore sought to extend and update our initial meta-analysis focused on the reduction of self-harm and suicide attempts, while also examining the effect of a variety of unique therapeutic interventions on levels of depressive symptoms and suicidal thoughts. In this way, we acknowledge the relevance of these mechanisms to the field and aim to advance our previous study findings with a wider criterion and selection of interventions. Our primary outcomes were the reduction in self-harm including NSSI or suicide attempts (SAs), and our secondary outcomes were the reduction in suicidal ideation (SI), meaning thoughts and feelings related to suicide, as well as depressive symptoms (DS).

## Methods

### Eligibility and selection

We followed the same methodology as our previous systematic review [see (11)], using “self-harm” as an encompassing term, including previous suicide attempts, non-suicidal self-injury, and deliberate self-harm with undetermined intent. However, in addition to the original systematic review's reduction of self-harm and suicide attempts (primary outcomes) we also examined decrease of suicidal ideation and depressive symptoms (secondary outcomes) as markers of the efficacy of the therapeutic interventions.

### Inclusion criteria

Inclusion to the update depended on: study type, sample age, and frequency of self-harm occurring within the sample. We included only studies which were clinical, randomized trials of therapeutic interventions, defined as any theoretically coherent, manualized, psychological, psychosocial, or pharmacological intervention, compared to a placebo or control treatment (11). Further, we included only studies with a majority (>50%) child and adolescent population (< 18 years old), engaged in either self-harm or had attempted suicide. Studies from all countries and languages were considered eligible, if they were accompanied by an English abstract.

### Exclusion criteria

Potential studies were excluded from the update if self-harm was a symptom of an overarching developmental condition (i.e., autism or intellectual disability). Finally, studies that did not meet the threshold score of >2 on the Jadad quality assessment tool (20), specifically used to ascertain methodological quality, coherence to blinding and allocation procedures, and amenability to participant attrition, were excluded from consideration.

### Literature search strategy

A literature search was conducted through standard online databases (OVID Medline, psycINFO, PubMed, EMBASE, and Cochrane Library) in order to identify RCTs evaluating the efficacy of therapeutic interventions for adolescents with self-harm against control conditions. To maintain consistency with our first study, the same inclusion and exclusion criteria were utilized in this update. We excluded studies in which self-harm occurred as a result of stereotypic self-injurious behaviors such as those seen in moderate to severe forms of neurological disorders (e.g., Autism, Intellectual Disability), due to the complex neurological mechanisms which underlie the self-harm not otherwise seen outside of these conditions. All the aforementioned databases were searched from the first available article until October 22^nd^, 2017. Consistent with our original methodology, the following subject headings or MeSH keywords were used: *self-injurious behavior; self-mutilation; suicide, attempted; suicide; drug overdose*. When available, filters for study type and participant age were applied, with an additional manualized filter used for psycINFO to specifically identify clinical trials.

## Results

### Study selection

**Figure 1:**
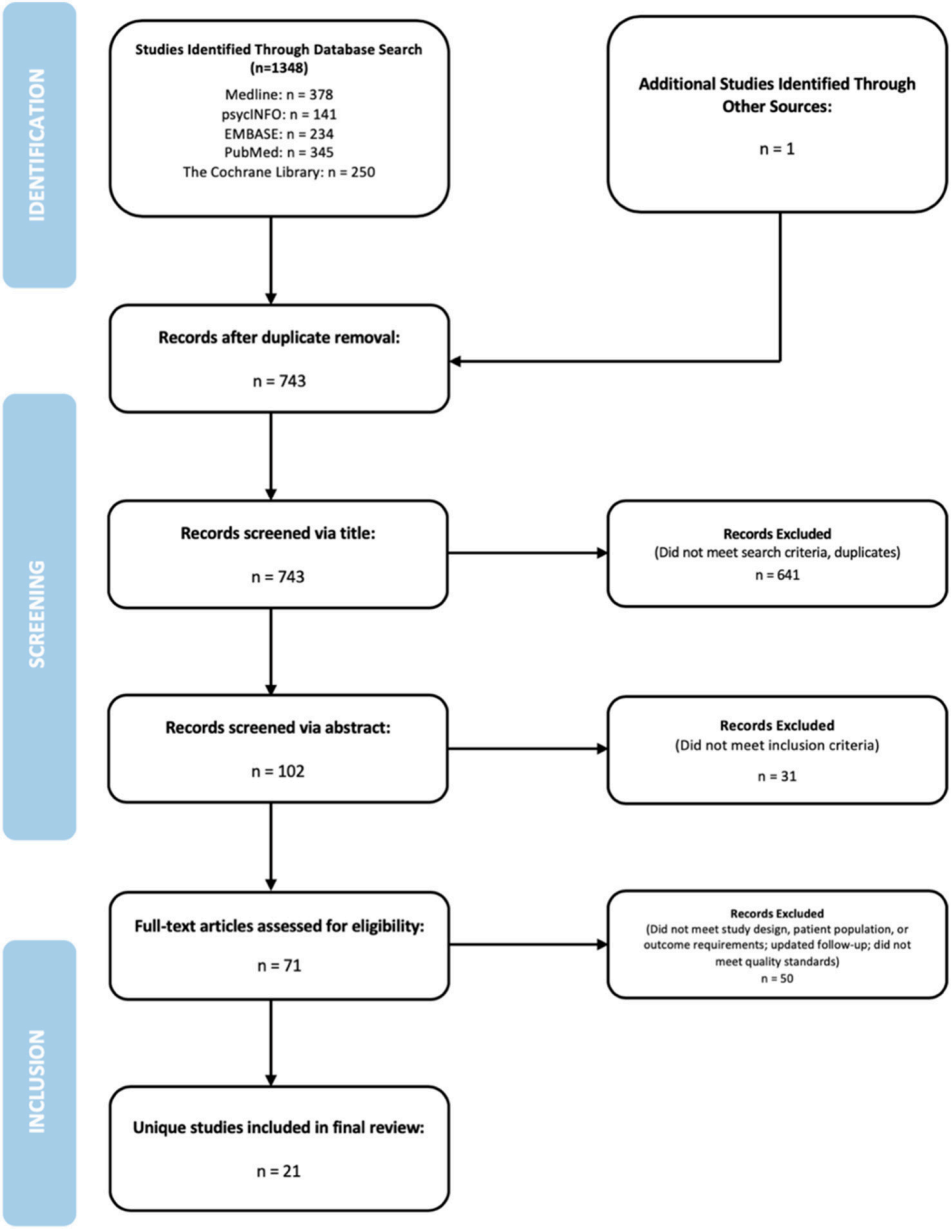
Study flow diagram.

A total of 1,348 articles were found, with 743 of those studies eligible for review following duplicate removal (Figure 1). The screening procedure consisted of three phases: title, abstract and full text screening, with the latter two conducted independently by the two authors (UI and NS). Title screening was conducted by NS as a preliminary measure to ensure the exclusion of any not-pertinent studies and duplicates, reducing the number of eligible studies from 743 to 102. Within the abstract screening phase, 31 articles were eliminated as they failed to meet any one aspect of the inclusion criteria, producing a yield of 71 articles for full text screening. In the third and final phase, we excluded 50 studies, 16 of which had a sample with a mean age above 18 years, 23 which did not have a majority of adolescents who engaged in self-harming behaviors, 9 which were not RCTs, 1 which has been replaced by a paper with more recent follow-up data, and 1 which did not meet quality standards. We chose to remove one study included in the original systematic review (21), as its investigation of a treatment designed to increase linkage to outpatient services did not assess the same outcomes which we are addressing in this update. Disagreements in any phase of the screening process were resolved by consensus discussion between the authors (UI and NS).

### Therapeutic interventions

The final selection of 21 studies contained 18 unique therapeutic interventions. Two of the final 21 studies (22, 23) were replication trials assessing the efficacy of Developmental Group Psychotherapy (24), while another paper (25) was a follow-up to a previous pilot trial of Emotion Training Regulation (ERT) (26). As such, 18 unique interventions were identified among all studies. To facilitate analysis, the interventions can be stratified and evaluated by the underlying theoretical principles, including self-driven cognitive, behavioral and regulatory interventions (referred to as self-driven) and interventions which require engagement with family and social support (referred to as socially-driven). A brief description of the interventions and the study origins are listed in Table 1, with self-driven and socially-driven components identified for each respective study in Table 2. Results below are reported first by interventions which we believed had a primarily self-driven focus, followed by those with a primarily socially-driven focus, and finally, interventions which demonstrated aspects of both self-driven and socially-driven principles.

**Table 1:** Descriptions and Study Origins of Therapeutic Interventions of the Selected Randomized Controlled Trials.

**TI name**	**Study origin**	**Description**
CBT-SP	Alavi et al. (27)	A 3-phase Cognitive-Behavioral Therapy protocol adapted specifically for suicide prevention. Utilizes cognitive behavioral principles according to the Stanley et al. model, and is comprised of 12 weekly sessions, the first of which includes parents.
Integrated Cognitive Behavioral Therapy (I-CBT)	Esposito-Smythers et al. (28)	Utilizes cognitive behavioral techniques such as restructuring, problem-solving, affect regulation and communication skills to remediate maladaptive cognitions and provide skills training for the attending adolescent and parents. One-year long intervention consisting of three treatment phases, involving with weekly, bi-weekly, and monthly individual adolescent, family, and parent training sessions.
DBT-A	Mehlum et al. (29)	Streamlined Dialectical Behavioral Therapy protocol adapted for adolescents, incorporating a new skills module to address emotion dysregulation amongst adolescents and their families. Nineteen-week long intervention involving weekly individual therapy, multi-family skills training, and family therapy sessions.
Developmental Group Psychotherapy	Wood et al. (24)	Integrates techniques from problem solving and cognitive and dialectical behavioral interventions to resolve issues around relationships, school problems, peer relationships, depression and self-harm, hopelessness, and feelings about the future. Delivered through a maximum of 19 acute and long-term group sessions run in tandem, administered by a variety of trained personnel.
Skills-Based Treatment (SBT)	Donaldson et al. (30)	Intervention designed to target problem solving and affect management skills in self-harming adolescents through cognitive behavioral strategies such as restructuring and relaxation. Delivered by therapists trained in SBT for an undefined number of sessions (mean number of sessions: 9.25).
Emotion Regulation Training (ERT)	Donaldson et al. (26)	Designed to teach participants ways of coping with affective instability, daily stressors, and psychological vulnerability through psychoeducation and behavior modification. Treatment is conducted through 17 weekly multi-phase group sessions delivered by therapists trained in ERT.
Safe Alternative for Teens and Youth (SAFETY)	Asarnow et al. (31)	Emergency Department (ED) family-centered intervention informed by CBT and DBT aimed to reduce future suicide attempts by strengthening protective supports, teaching skills for managing stress reactions and formulating strategies for creating a safe environment for the adolescent. Treatment is conducted through 12 weekly individual and joint sessions for adolescents and their parents, delivered by two therapists, each working with the family.
Mentalization Based Therapy for Adolescents (MBT-A)	Rossouw and Fonagy (32)	An adapted form of Mentalization-based Treatment, a manualised intervention focusing on impulsivity and affect regulation, helping to enhance the patient's understanding of how to represent feelings in emotionally challenging situations. Year-long intervention with weekly individual and family based therapy (MBT-F) delivered by trained therapists.
Cognitive Analytic Therapy (CAT)	Chanen et al. (33)	Time limited, integrated model of development and psychopathology, equipping the patient with tools more effectively manage stressful situations which could lead to a repetition of pathological behavior. Conducted through 24 weekly sessions delivered by therapists trained in CAT.
Therapeutic Assessment	Ougrin et al. (34)	Manualised assessment protocol for self-harming adolescents facilitating the identification of the target problem, enhancing motivation for change, and exploring ways of relieving vicious cycles. Assessment takes place in one session and is delivered by a trained clinician.
Emergency Tokens	Cotgrove et al. (35)	Self-harming adolescents were allotted a token allowing hospital re-entry without question, to be used when adolescent was in need of escaping an intolerable (family) environment.
Home-based Family Intervention	Harrington et al. (36)	Short-term, intensive, focused, action orientated intervention used to address family dysfunction without lengthy treatment commitments or the need to present to a hospital setting. Conducted by psychiatric social workers during 5 home-based therapy sessions.
Family Intervention for Suicide Prevention (FISP)	Asarnow et al. (6)	Brief ED intervention which focuses on building a collaboration between adolescents and their parents by identifying and addressing the causes, reaction, and future actions related to the committed suicide attempt. Administered by a trained clinician.
Family-Based Crisis Intervention	Wharff et al. (37)	Brief intervention which provides the family with tools to manage current and future crises through psycho-education, cognitive behavioral skill building, therapeutic readiness and safety planning. Delivered during the adolescent's visit to the ED by a research clinician.
Attachment-Based Family Therapy (ABFT)	Diamond et al. (38)	Designed to improve problem solving, affect regulation and organization within the family. Number of sessions vary, depending on the adolescent's progress in resolving 5 specific tasks, and is delivered by a therapist trained in ABFT.
Youth Nominated Support Team-I	King et al. (39)	Supplements routine care by facilitating weekly contact between adolescents and their chosen support person (outside of the family), based on the notion that support people may minimize the impact of negative family environment. Support is provided to their adolescent by their nominated individual, who is asked to be in weekly contact with the adolescent. Support personnel are given 1.5–2 h of training.
Youth Nominated Support Team-II	King et al. (40)	Similar to YST-I, but with updated psychoeducation materials and the requirement that the nominated support person be an adult (rather than a peer). The support person has weekly check-ins with the adolescents for 3 months following hospitalization.
Resourceful Adolescent Parent Program (RAP-P)	Pineda and Dadds (41)	Strengths-based family psycho-education program, enhancing understanding of SH and SA, along with strategies to help minimize future self-injurious behavior, and information to facilitate access to support services. Sessions 2 h and held once a week or fortnightly.

**Table 2:** Types of Therapeutic Intervention for the Selected Randomized Controlled Trials and Aspects of Individual or Social Components.

	**Self-driven Components**					**Social Components**			
	**CBT**	**DBT**	**MBT**	**CAT**	**Problem Solving**	**Social Support**	**Family Involvement**	**Psycho-education**	**Communication skills**
Alavi et al. (27)	•							
Asarnow et al. (6)							•	
Asarnow et al. (31)	•	•					•	
Cotgrove et al. (35)								
Chanen et al. (33)				•				
Diamond et al. (38)							•	
Diamond et al. (30)	•				•		•	
Esposito-Smythers et al. (28)	•						•		•
Green et al. (23)	•	•					•		•
Harrington et al. (36)							•	
Hazell et al. (22)	•	•			•				•
King et al. (39)						•		•
King et al. (40)						•		•
Mehlum et al. (42)		•					•		•
Ougrin et al. (34)				•			•	
Pineda and Dadds (41)							•	•
Rossouw et al. (32)			•				•	
Schuppert et al. (26)		•						
Schuppert et al. (25)		•						
Wharff et al. (37)	•						•	
Wood et al. (24)	•	•			•				•

### Primary outcome assessment

Table 3 provides a brief summary of primary outcomes of the studies for comparison of therapeutic intervention examined, and those that had significant group differences, significant overall differences, or null or negative findings. Of the 21 studies examined, 16 studies explored primary outcomes, out which 5 (31.3%) found significant group differences for intervention vs. treatment as usual (24, 28, 31, 32, 42), across all types of treatments.

**Table 3:** Selection of Randomized Control Trials and Results Investigating the Efficacy of Therapeutic Interventions (TI's) Versus Control Treatments on Self-Harming Adolescents.

**Study**	**Sample Criteria (after attrition)**		**Inclusion Criteria (Self-harm definition)**	**Relevant Diagnostic Tools**	**Outcomes Investigated**			**Treatment Type**		**Periods of Assessment**	**Outcomes and Findings**
	**Treatment/Control (n)**	**Demographics (mean age)**			**Self-Harm**	**Suicidal Ideation**	**Depressive Symptoms**	**Intervention**	**Control**	
Alavi et al. (27) Iran	15/15	12–18 (16.1) 90% female	**Hospital presentation following suicide attempt within 3 months of study**	Scale for Suicidal Ideation (SSI) Hopelessness Inventory (BHI) Depression Inventory (BDI)		✓	✓	CBT for suicidal ideation	WL	12 weeks	Significant reduction in suicidal ideation, hopelessness, and depressive symptoms in treatment group
Asaranow et al. (6) USA	89/92	10–17 (14.7) 69% female	**Hospital presentation following suicide attempt or suicidal ideation** (intentional self-injury with/without intent to die)	Suicide attempts on the NIMH-DISC-IV Center for Epidemiological Studies Depression Scale (CES-D)	✓		✓	Family Intervention for Suicide Prevention (FISP)	TAU	~2 months	No significant reduction in self-harm or depressive symptoms, but improved linkage to outpatient care
Asarnow et al. (31) USA	20/22	11–18 (14.62) 88% female	**Suicide attempt within 3 months of study** or ≥**3 episodes of self-harm within lifetime**	Columbia-Suicide Severity Rating Scale (C-SSRC) NIMH-DISC-IV Suicide History Interview (SHI) Service assessment for children and Adolescents (SACA)	✓			SAFETY (DBT informed CBT)	E-TAU	3 months	Significant differences between groups for SA at 30month timepoint, no difference between groups on NSSI.
Chanen et al. (33) Australia	44/42	15–18 (16.4) 76% female	**Fulfillment of 2/9 DSM-IV Criteria for Borderline Personality Disorder**	Semi-structured interview for paras-suicidal behavior developed by research group	✓			Cognitive Analytic Therapy	GCC	6, 12, 24 months	Reduction of parasuicidal behaviors seen within whole cohort, with no significant group differences
Cotgrove et al. (35) UK	47/58	≤ 16 (14.9) 85% female	**Hospital presentation following suicide attempt** (attempted suicide, deliberate acts of self-injury or self-poisoning)	Clinical records Questionnaire, unspecified		✓		Token for readmission to hospital + AAU	AAU	12 months	Fewer people (50%) with hospital readmission tokens re-attempt suicide than those without, but no significant effect found within treatment group
Diamond et al. (38) USA	35/31	12–17 (15.1) 83% female	>**31 SIQ score** >**20 BDI-II**	Suicide Ideation Questionnaire (SIQ-JR) Beck Depression Inventory (BDI-II) Scale for Suicidal Ideation (SSI)		✓	✓	Attachment-based Family Therapy	E-TAU	6, 12, 24 weeks	Significant reduction in SI within treatment group at all-time points; Significant reduction in DS within treatment group my mid-treatment, but loss of effect at post-treatment and follow-up
Donaldson et al. (38) USA	21/18	12–17 (15) 82%female	**Hospital presentation following suicide attempt** (intentional non-fatal self-injury with intent to die)	Structured Follow-up Interviews SIQ CES-D	✓	✓	✓	Skills-Based Treatment (SBT)	SRT	3, 6 months	Overall reduction in likelihood of re-attempting suicide and an improvement in ideation and depressive symptoms, but no significant differences between groups at any point
Esposito-Smythers et al. (28) USA	20/20	13–17(15) 68% female	**Suicide attempt within 3 months of study or** ≥**41 on SIQ in the past month**	SIQ Columbia Impairment Scale (CIS) Reynolds Adolescent Depression Scale (RADS-2)	✓	✓	✓	I-CBT	E-TAU	18 months	Significant reduction in suicide attempts in treatment condition; overall improvement in cohort on ideation and depressive symptoms with no significant differences between groups
Green et al. (23) UK	179/180	12–17 89% female	**Hospital presentation following** ≥**2 episodes of self-harm within 12 months** (intentional self-inflicted injuries or overdose of toxic substances)	SIQ Mood and Feeling Questionnaire (MFQ) Health of Nation Outcomes Scales for Children and Adolescents (HoNOSCA)	✓	✓	✓	Developmental Group Psychotherapy	TAU	6,12 months	Overall improvement within cohort on self-harm, ideation and depressive symptoms, but no significant differences between groups at any point
Harrington et al. (36) UK	85/77	≤ 16 (14.5) 90% female	**Hospital presentation following deliberate self-poisoning** (ingestion of substances not for human consumption, or overdose)	SIQ Hopelessness Questionnaire McMaster Family Assessment Device		✓		Home-based Family Intervention + TAU	TAU	2, 6 months	No significant differences between groups at any point on rates of suicidal ideation
Hazell et al. (22) Australia	35/37	12–16 (14.5) 91% female	≥**3 episodes of self-harm, one happening within 1 month of study** (intentional self-inflicted injury irrespective of intent)	SIQ MFQ Schedule for Affective Disorders and Schizophrenia (K-SADS) HoNOSCA	✓	✓	✓	Developmental Group Psychotherapy	TAU	2, 6, 12 months	Overall improvement within cohort on self-harm, ideation, and depressive symptoms but no significant differences between by follow-up; Significantly higher proportion of treatment group engaged in self-harm until 6 months
King et al. (39) USA	113/123	12–17 (15.3) 68% female	**Significant suicidal ideation or suicide attempt with 1 month of study / score of 20 or 30 on Self-harm subscale of the Child and Adolescent Functional Assessment Scale (CAFAS)**	SIQ-JR Spectrum of Suicide Behavior Scale Youth Self-Report (YSR) RADS CAFAS	✓		✓	YST-I + TAU	TAU	6 months	No significant difference in suicide attempts between groups. Small to medium effect on the reduction of suicidal ideation only after altering analyses from intent-to-treat to only in female participants
King et al. (40) USA	223/225	13–17 (15.6) 71% female	**Significant suicidal ideation or suicide attempt within 4 weeks of study**	SIQ-JR BHS Children's Depression Rating Scale Revised (CDRS-R)	✓		✓	YST-II + TAU	TAU	6 weeks, 3, 6, 12 months	No significant reduction in suicide attempts. Overall improvement on depressive symptoms (moderated by multiple attempts) lasting 6 weeks.
Mehlum et al. (42) Norway	39/38	(15.6) 83% female	≥**1 episode of self-harm within 16 weeks of study / Fulfillment of 2 criteria of BPD / fulfillment of 1** + **2 subthreshold criteria of BPD** (intention self-inflicted injury irrespective of intent)	Lifetime Parasuicide Count (LPC) Interview Suicide Intent Scale (SIS) SIQ-JR Short MFQ	✓	✓	✓	DBT-A	E-TAU	9, 15, 19, 71 weeks	Significant reduction in self-harm, ideation, and depressive symptoms at 19 weeks, but loss of significance at 1 year follow-up;
Ougrin et al. (34) UK	35/34	12–18 (15.5) 80% female	**Engaging in self-harm without prior involvement with psychiatric services** (intentional self-inflicted injury or self-poisoning irrespective of intent)	Health department records including: CAMHS, A&E, and Primary Care	✓			Therapeutic Assessment (TA)	AAU	24 months	No significant reduction in hospital presentations for self-harm, though treatment engagement increased significantly
Pineda and Dadds (41) Australia	22/18	12–17 (15.14) 75% female	≥ **1 episode of suicidal behavior** (suicidal ideation, intent, suicide attempt, self-injury) within the last 2 months before referral to hospital; residing with at least 1 parent	Adolescent Suicide Questionnaire- Revised (ASQ-R)	✓			RAP-P	Routine Care	3, 6 months	Significant improvement in suicidal behavior at 3 and 6 months in RAP-P group, compared to control group.
Rossouw et al. (32) UK	20/20	12–17 (14.7) 80% female	≥**1 episode of self-harm within past month** (intentional self-inflicted injury irrespective of intent)	Risk-Taking and Self-Harm Inventory (RTSHI) MFQ	✓		✓	MBT-A	TAU	3, 6, 9, 12 months	Significant reduction in self-harm and depressive symptoms for treatment group during treatment and at follow-up
Schuppert et al. (26) Holland	23/20	14–19(16.14) 88% female	**Fulfillment of 2/9 DSM-IV Criteria for Borderline Personality Disorder** including: Recurrent suicidal behavior, gestures, threats, or self-mutilation	Clinical interview Youth Self-Report (YSR) Internalizing & Externalizing	✓		✓	Emotion Regulation Training + TAU	TAU	3, 6 months	Reduction in self-harm and depressive symptoms seen within whole cohort with no significant group differences
Schuppert et al. (25) Holland	54/55	14–19 (15.98) 96% female	**Fulfillment of 2/9 DSM-IV Criteria for Borderline Personality Disorder** including: Recurrent suicidal behavior, gestures, threats, or self-mutilation	Clinical interview Youth Self-Report (YSR) Internalizing & Externalizing	✓		✓	Emotion Regulation Training + TAU	TAU	6, 12 months	Reduction in self-harm and depressive symptoms seen within whole cohort with no significant group differences (information obtained via e-mail)
Wharff et al. (37)USA	68/71	13–18 (15.5) 72% female	**Hospital presentation for suicidality** (suicidal self-identification, adult-noted suicidality, suicide attempt)	Reasons for Living Inventory for Adolescents (RFL-A)		✓		Family-Based Crisis Intervention	TAU	Post-test, 3 day, 1 week, 1 month	Overall reduction of ideation and depressive symptoms within whole cohort with no significant group differences; Intervention group significantly less likely to be re-hospitalized post treatment
Wood et al. (24) UK	32/31	12–16 (14.25) 78% female	**Hospital presentation following incident of self-harm** (intentional self-inflicted injury irrespective of intent)	MFQ SIQ HoNOSCA	✓	✓	✓	Developmental Group Psychotherapy	TAU	7 months	Significant reduction in likelihood of re-attempting suicide within treatment group; overall improvement within cohort but no treatment effect on ideation and depressive symptoms

*TAU, Treatment As Usual; E-TAU, Enhanced Treatment As Usual; GCC, Good Clinical Care; AAU, Assessment As Usual; SDP, Standard Disposition Planning; SRT, Supportive Relationship Treatment; I-CBT, Integrated CBT*.

#### Significant differences in therapeutic interventions vs. treatment as usual

We first examined interventions that addressed individual problem solving, mentalization, cognitive behavior or skills deficits [these included treatments such as Cognitive Behavioral Therapy [CBT], Mentalization Based Therapy [MBT], Dialectical Behavioral Therapy [DBT]]. The only identified studies with a purely self-driven intervention model were those that evaluated Developmental Group Psychotherapy, an intervention which used cognitive-behavioral, problem-solving, dialectical, and psychodynamic group psychotherapy strategies. In a small initial trial among adolescents with repeated SH referred to child and adolescent mental health services in the UK, Developmental Group Psychotherapy compared to treatment as usual was associated with a significantly lower risk of repeating self-harm, with a lower latency period for repeated attempts, indicating an absolute risk reduction of 26% (24). The authors cautioned that this strong effect was likely due to a smaller sample, and urged replication studies with a larger sample. Indeed, two efforts at replication failed to find a significant advantage for the Developmental Group Psychotherapy intervention. These trials included one conducted in Australia with supervision from the original UK development team and somewhat different sampling criteria: youths referred for general child and adolescent mental health services identified with repeat self-harm (22). The other trial was a large trial (*N* = 366) conducted in the UK with members of the original development team and also failed to find an advantage for the Developmental Group Psychotherapy (23) over treatment as usual.

One study conducted in Australian outpatient mental health clinics used a socially driven intervention to evaluate a strength-based family education program, called Resourceful Adolescent Parent Program (RAP-P). Among patients recruited suicidal adolescents from emergency departments or public mental health services, the RAP-P program resulted in a significant improvement on a 9-item suicide index assessing suicide ideations, plans, threats, self-harm, and suicide attempts both at the 3-month post-treatment point, and at a 6-month follow-up compared to treatment as usual (41). This study was the only exclusively socially-driven intervention model to find a significant group difference on primary outcomes and it should be noted that the outcome variable was a broad measure of suicidality rather than a measure specifically of self-harm or suicide attempts.

Four trials examined more combined self-driven and system driven approaches. We begin with Mentalization-Based Treatment for adolescents (MBT-A), a manualized one-year psychodynamic psychotherapy rooted in attachment theory. MBT-A has a strong self-driven component consisting of weekly individual MBT-A sessions and compared to the other approaches in the combined approach group, the weakest of the socially-driven components; specifically, monthly family Mentalization-based family therapy. In an initial trial, Rossouw and Fonagy (32) found that adolescents selected for the presence of both self-harming behaviors and depression in the MBT- A condition had fewer self-harm episodes over the course of the treatment compared to treatment as usual youths and that the MBT-A group had a higher recovery rate and a reduction of self-harm at the end of the 12-month treatment. Thus, results from this initial trial support the efficacy of MBT-A for reducing overall self-harm. Results specifically for suicidal behavior were not reported.

Dialectical Behavioral Therapy for Adolescents, (DBT-A) addresses self-driven cognitive-behavioral and regulatory processes (e.g., emotion regulation, distress tolerance, and interpersonal effectiveness) and the social environment through inclusion of a weekly multi-family skills training group and as needed family therapy sessions. A first RCT evaluating DBT-A was conducted in Norwegian clinics and recruited youths with both at least two episodes of self-harm and symptoms of borderline personality disorder (43). This trial compared DBT-A to treatment as usual enhanced by a therapist agreement to provide at least 1 weekly session during the trial. Results indicated that the 19-weeks DBT-A reduced the frequency of self-harm with large effect sizes, compared to moderate and weak effect sizes in the TAU condition (29). The advantage of DBT-A for reducing self-harm extended to a 1-year post-treatment follow-up, with DBT-A youths continuing to demonstrate fewer episodes of self-harm compared to treatment as usual youths (42). The authors looked at a range of additional clinical outcomes including suicidal ideation, hopelessness, depression, borderline symptoms, and global functioning. Results indicated that while there was an initial advantage for DBT-A and DBT-A youths continued to show improved clinical and functioning outcomes at the 1-year post-treatment follow-up, with time the TAU youths caught up and looked similar to the DBT-A youths on these more general outcomes. This trial was not powered to evaluate outcomes regarding suicide attempts, thus such data were not reported.

The combined self-driven and socially-driven intervention model was used in two U.S. treatment development trials. Both trials used a 2-therapist model with one therapist focusing on the youth and the other the parent, and both studies showed evidence of benefits in reducing the risk of suicide attempts. First, Esposito-Smythers et al. (28) tested an integrated CBT (I-CBT) protocol for suicidality (along with co-occurring alcohol and drug related problems) and found that those randomized to I-CBT had fewer suicide attempts over the course of 18-months compared to those in the control condition. Second, Asarnow et al. (31) developed a DBT-informed cognitive-behavioral family treatment (referred to as SAFETY) which included attention to strengthening self-driven cognitive, behavioral and regulatory processes in the youth and parents, and family sessions aimed at promoting increased support and protection within the family and broader social environment. Results of this trial indicated a statistically significant advantage for SAFETY in decreasing suicide attempts over the 3-month treatment period, and reducing the risk of a first incident suicide attempt over a 6 to 12-month follow-up period. Weaker non-significant group differences were found for non-suicidal self-injury. While results of these trials are encouraging, it should be noted that both studies were relatively small treatment development trials, underscoring the need for cautious interpretation until replication is achieved.

#### Overall group differences irrespective of therapeutic intervention

Overall symptom reduction across both treatment and control groups was found throughout several other studies included in this review, not specific to intervention type, including the Green et al. (23) trial of Developmental Group Psychotherapy intervention. In a specific clinical sample of Borderline Personality Disorder (BPD), three studies assessed the efficacy of cognitively-informed interventions. Chanen et al. (33) and Schuppert et al. (25, 26) investigated the efficacy of Cognitive Analytic Therapy (CAT) and Emotion Regulation Training (ERT), respectively, against TAU. Both CAT and ERT emphasize ways to react and respond to stressful situations with tools to more effectively manage stressful situations with aspects of self-driven processes. Both studies only observed the reduction of self-harm within the whole cohort with no statistically significant group differences, indicating that these therapies do not appear to perform any better than TAU in reducing self-harm within Borderline patients.

#### Null or negative findings

Several interventions included in this update demonstrated non-significant or negative findings. For instance, The Youth-Nominated Support Treatment (YST-I) is a socially driven treatment that focuses on improving support in youths' social support network and is added to TAU, with version II of the intervention focused on strengthening social support in youth-nominated supportive adults, rather than adults and peers. In both YST-I (39) and YST-II (40), no significant treatment effect was found in reduction of SA; authors emphasized the need for further research using this mode.

Cotgrove et al. (35) demonstrated non-significant findings regarding secondary prevention of suicide attempts in adolescents, examining re-admissions to Emergency Rooms. Adolescents were randomly allocated to a group receiving tokens guaranteeing re-admission to emergency services if they felt unable to cope within their environment. While there was no significant difference noted between adolescents with tokens and those without, those in the treatment condition had fewer repeat attempts than the control group, suggesting possible efficacy of a secondary prevention mechanism.

Two studies identified advantages of brief mental health interventions for linking youths to outpatient treatment after emergency presentation for suicidality and/or self-harm. Asarnow et al. (6) looked at a brief, cognitive-behavioral family-based Emergency Department (ED) intervention and found an advantage of the this intervention compared to treatment as usual for establishing linkage to outpatient care (the primary study outcome). Clinical outcomes were not evaluated close in time to the Emergency Department intervention. However, when clinical outcomes were evaluated roughly 2 months after discharge from the Emergency Department, no statistically significant advantage was found for this intervention in reducing suicide attempts. Importantly, there was also no evidence that linkage to outpatient treatment as usual after discharge from emergency services had any advantages relative to no post-discharge community treatment as usual.

Ougrin et al. (34) applied a brief therapeutic intervention incorporating elements of cognitive analytic therapy called Therapeutic Assessment (TA) following emergency presentation of self-harm. While there was no significant difference in the frequency of self-harm resulting in emergency presentations between the Therapeutic Assessment and treatment as usual groups, overall treatment engagement remained higher in the Therapeutic Assessment group than the control group. Collectively, the Asarnow et al. & Ougrin et al. studies underscore the value of brief mental health interventions for improving linkage to outpatient treatment after emergency presentation for self-harm and suicidality, as well as the importance of efforts to identify effective treatment strategies and implement them effectively within community programs.

### Secondary outcome assessment: suicidal ideation, depression, other clinical, and functioning outcomes

#### Significant differences in therapeutic interventions vs. treatment as usual

Of the possible 21 studies, 17 studies explored secondary outcomes of depressive symptoms and suicidal ideation, of which 5 (29.4%) yielded significant differences by the intervention. Beginning with studies using primarily self-driven interventions, MBT-A was found to yield a significant reduction in depressive symptoms at the 12-month point (32). Both the treatment and control group showed reduced depressive symptoms, and a significant reduction over time was seen only in MBT-A youths condition. The largest mean difference between groups was seen at 9 months. Using a 12-week treatment period, Alavi et al. (27) evaluated the CBT-Suicide Prevention (CBT-SP) treatment developed for the Treatment of Adolescent Suicide Attempter's (TASA) study (44), results indicated significant reductions in both suicidal ideation and depressive symptoms at the end of the 3-month treatment period. These results are similar to those from the TASA trial, which was originally designed as an RCT but random assignment was discontinued due to patients' reluctance to accept randomization. The TASA trial found relatively low rates of suicidal events, including suicide attempts, interrupted suicide attempts, and levels of suicidal ideation requiring emergency evaluation or hospitalization (19) as well as declines in suicidal ideation and depressive symptoms over the 6-month treatment period (45).

For interventions focusing on social support and psychoeducation strategies, King's investigations of both versions of the Youth-Nominated Support Team intervention decreased suicidal ideation over time in both groups. Further, the first version of the intervention, YST-I, allowed both youth-nominated peers and adults in the Support Team and demonstrated a small to medium effect on the reduction of suicidal ideation only after altering analyses from intent-to-treat to actually treated; an effect that was seen only in female participants (39). Following with a study of the adapted YST-II intervention, which focused on youth-nominated adults, King again demonstrated a small to medium effect in the reduction of suicidal ideation, this time, only in those participants who had a history of multiple suicide attempts (40).

Another socially-driven intervention, Attachment-Based Family Therapy (ABFT), compared to TAU, was shown to have very strong effects in reducing suicidal ideation during all points of treatment with the strongest effect observed at 24 weeks, the final follow-up (38). This effect was seen within the total sample, and within a subsample of adolescents who met the diagnostic criteria for clinical depression. Additionally, a significant effect in the reduction of suicidal ideation was seen within the Home-Based Family intervention, but only when controlling for depression.

#### Overall group differences irrespective of therapeutic intervention

Reductions over time in suicidal ideation were observed in nearly all studies, including those evaluating mentalization treatment, DBT-A, integrated CBT for suicidality and substance abuse, skills based treatment, youth-nominated support teams, and attachment based family treatment (28–30, 32, 38).

While a significant reduction in ideation was observed in 3 studies based on social or family models (38–40), none of these studies identified significant differences in the reduction of depressive symptoms between treatment and control groups at the final follow-up measure. The Attachment-Based Family therapy intervention appeared close to producing a nearly significant result at the 6 and 12-week measurements, but was unable to reach statistical significance altogether at the end of the trial (38).

When examining socially-driven interventions within an emergency service settings, Asarnow et al. (6) did not examine suicidal ideation or depressive symptoms close in time to the emergency intervention. However, when followed up roughly 2-months post -hospital discharge there were significant declines in depressive symptoms across groups. Of the socially-driven interventions administered during a presentation to emergency services or at the time of a psychiatric assessment, only Wharff investigated whether a Family-Based Crisis Intervention, a multi-module single session intervention would impact the adolescent's suicidal ideation (46). While an overall reduction in suicidal ideation was seen within the cohort, there was no significant treatment effect. In contrast to the other studies included this review, Wharff conducted pre-and post-test measures a mere 4–h apart.

#### Null or negative findings

Despite noting significant group differences in primary outcomes, Wood et al. (24) found no significant effect of Developmental Group Psychotherapy on reducing depressive symptoms and suicidal ideation. Consistent with what was noted for primary outcomes, neither of the two additional trials evaluating the same treatment (22, 23) saw treatment effects in secondary outcomes of reducing suicidal ideation or depressive symptoms.

Asarnow et al. (6) found no statistically significant advantage of the Emergency intervention in reducing depression levels, even though the intervention was associated with improved linkage to outpatient mental health services was observed. Indeed, there was no evidence that attendance in outpatient community treatment as usual was associated with lower depressive symptoms or suicidality.

## Discussion

We set out to examine the available literature for adolescents with a recent history of self-harm or suicide attempt, with the overall aim of clarifying which therapeutic interventions and approaches show evidence of benefits for reducing self-harm, suicide attempts, as well as suicidal ideation and depressive symptoms. Of the 18 unique interventions identified through this review, treatments that target individual, self-driven (cognitive-behavioral, self-regulatory processes) and socially-driven (family or social support network) processes appeared to show the greatest promise for reducing suicide attempts (28, 31), and there are data supporting the benefits of DBT-A and MBT-A (combined self-driven and systems-driven approaches), for reducing overall self-harm. If the somewhat different variations of CBT are considered together, and DBT-A is classified as a type of CBT, CBT is the only intervention type where initial positive findings have been replicated independently. It should be noted, however, that all of the CBT interventions with evidence for efficacy have strong family systems-driven components (I-CBT, SAFETY, DBT-A). Other interventions with initial positive outcomes, such as MBT-A require testing in adequately powered trials and replication.

The results of this review update are demonstrative of the effectiveness of DBT-A and CBT, the only interventions where initial positive findings have been replicated independently. These interventions are therefore an invaluable part of the clinical treatment of young people who present with self-harm and a history of suicide attempts.

Turning to the secondary outcomes of suicidal ideation and general measures of suicide risk, current research supports the efficacy of attachment based family treatment and the Resourceful Adolescent Parent Program. Results were not reported on suicide attempts specifically or self-harm in either trial, and replication is needed. While not all studies have reported on suicidal ideation when suicide attempts and self-harm were primary outcomes, both MBT-A and DBT-A have shown significant advantages in reducing both self-harm and suicidal ideation. Rossouw and Fonagy (32) reported an advantage for MBT-A compared to treatment as usual at end of the year-long treatment. Mehlum et al. (42) similarly reported an advantage for DBT relative to treatment as usual at end of treatment, which was at 16 weeks, although treatment as usual youths had caught up with the DBT-A youths by 71-weeks.

Results on depression outcomes tended to be similar to those for suicidal ideation, with a tendency for depression levels to diminish over time, and between group differences observed in the studies and time points where benefits on suicidal ideation were observed. The two studies that evaluated borderline symptoms (DBT-A and MBT-A) reported intervention benefits in reducing symptoms of Borderline Personality Disorder. Both suicidal ideation and depressive symptoms have been shown to precipitate non-suicidal self-injury and suicide attempts in adolescents (19, 47), and thus should not be overlooked as integral symptoms to address during treatment in future research.

Overall, the studies which showed significant effects in the reduction of outcomes at any point during treatment (without adjustments) were similar in several characteristics. First, these interventions mandated the family or support person's involvement in the adolescent's therapeutic journey in addition to the adolescent's individual therapy; five interventions included parental involvement throughout the duration of the intervention, through family training, family therapy, and/or family planning (28, 29, 31, 32, 38, 41). The Responsible Adolescent Parenting Program (2013) was the only exclusively socially-driven parenting intervention that yielded significant effects, suggesting a need for replication studies using this treatment. Finally, we noted that the effective interventions all share emotion regulation, problem solving, and communication skills as key tenets of the intervention. While dysregulated affect is shown to be a predictor of suicide attempts and non-suicidal self-injury (47), subsequent research is needed to assess whether problem-solving and communication skills would contribute to the reduction of self-harm, suicidal ideation, or depressive symptoms.

The current National Institute for Health and Care Excellence (NICE) guidelines (48) for the treatment of adolescents with self-harm, suggest tailored treatments incorporating elements of cognitive behavioral, psychodynamic and problem-solving therapies. Results of this review are generally consistent with these guidelines, and clinical guidance is needed to support optimal clinical care for this potentially life-threatening problem. However, it is important to note that the evidence is limited. We still lack replicated evidence of treatment efficacy for any of the reviewed interventions. It is also important to note that the sampling protocols and populations differed across studies, and these sampling differences could lead to differences in treatment efficacy and study results. For instance, the DBT-A trial selected youths based on the presence of repeated self-harm and symptoms of borderline personality disorder, the MBT-A trial selected youths for the presence of self-harm within the past month, the Integrated CBT model recruited youths with both suicidality (attempts or ideation) and substance abuse, the SAFETY trial selected youths based on the presence of suicide attempts or repeated self-harm, and the Peer Nominated Support Team trials recruited youths with previous suicide attempts or suicidal ideation. Because trials were conducted across different nations and health systems, “treatment as usual” will have varied considerably, and this could conceivably have affected the observed between group differences. Further, with few exceptions (32, 41) studies have not yet reported on treatment mediators and studies aimed at treatment mechanisms associated with reduced fatal and non-fatal self-harm risk would help guide the field. Research focusing on targeted interventions such as those aimed at reducing access to dangerous methods of self-harm (firearms, poisoning) could also help inform clinical care and data are accumulating supporting the value of such interventions.

### Strengths and limitations

The current review supplements the literature by conducting a systematic review on not only suicide attempts and self-harm, but also addresses the links between suicidal ideation and depressive symptoms in adolescents. While we were able to add a more comprehensive component to the systematic review, due to the complexity of the varied studies and primary and secondary outcomes, additional research is needed to identify effective treatment strategies, provide guidance regarding how to best personalize treatment and match youths and families to treatments that are most likely to be beneficial, and to develop cost-effective treatment delivery strategies. There are no published RCTs of pharmacological interventions for the reduction of self-harm. However, many young people who self-harm are offered pharmacological treatment to address co-occurring psychiatric symptoms. While an investigation of the possible influence of pharmacological interventions was out of the scope of the current review, future work addressing both psychosocial and psychopharmacological elements of treatment is needed. To date, there have been no RCTs evaluating the efficacy of psychopharmacological treatments for reducing self-harm, though this may also be a potential avenue for future research. In following the methodology of the previous analysis, studies which did not adhere to an RCT design were excluded, though may nonetheless be insightful in regards to reducing self-harm in young people. Additionally, as the search terms used to identify studies on self-harm were derived from the subject headings of the relevant databases and were limited to studies published in English, some studies representing unique cultural outlooks on self-harm may have been omitted. Indeed, the heterogeneity of the studies included in this review makes it difficult to account for additional influential factors, including the participants' previous engagement with therapy, their mental health histories and psychiatric co-morbidities, and the quantity and severity of their previous self-harm, among others. Future reviews may wish to pay particular attention to such elements in order to understand whether certain interventions work more effectively in some unique cases over others. A variety of additional factors, including small and highly selective samples limit the generalizability of the findings in some of the studies included in this review. Finally, the current failure to replicate certain interventions in different cultural contexts underscores the challenges for exporting treatment strategies across different cultures and settings, and the importance of building international consensus and developing care strategies that can work across diverse cultural contexts and health systems.

### Future directions for effective interventions

Considering future directions for studies aimed at decreasing self-harm and suicide attempts in adolescents, some treatments (such as DBT-A) led to significant reductions on outcomes more rapidly than others. Cost analyses could further inform knowledge about the viability of delivering certain interventions over others in routine clinical settings to large populations. Several of the evaluated interventions require multiple personnel (e.g., 2-therapist model used in the Integrated CBT model, the SAFETY intervention, and DBT-A with skills trainer and individual therapist). Likewise, whereas several of the interventions are completed in under 6 months, others require long and perhaps costly commitments to therapy, which may act as barriers to treatment adherence, particularly for adolescents who experience a lack of motivation to attend sessions as a symptom of depression (49, 50). These cost considerations will also have a major bearing on the likelihood of interventions being implemented in routine health setting. Additionally, some of the multi-component treatments, such as DBT-A which requires 1 h weekly psychotherapy plus 2 h of multi-family skills group may require a more intensive and burdensome treatment dose than needed for some youths and families. Stepped care approaches that match treatment intensity to assessed level of risk and need may prove helpful for identifying the most cost-effective treatment delivery strategies.

The results of a large, multi-center RCT investigating the effectiveness of Family Therapy in reducing self-harm in adolescents (51) were published following the intial literature review, and thus cannot be included in the results of the current update. However, these results, which demonstrated that Family Therapy was more costly and no more beneficial than treatment as usual, are nonetheless an important contribution to understanding which interventions are viable and effective in reducing self-harm in young people. In comparison to other therapies which include a strong family-based component as part of the treatment (such as DBT-A, MBT-A, and RAP-P among others), participants in the Family Therapy group were provided with 6–8 monthly sessions of therapy; far fewer than those participants which received any of the aforementioned treatments, indicating that the duration and intensity of the treatment may be an important factor in the success of an intervention aiming to reduce self-harm and preventing suicide attempts.

Other RCTs published following the completion of our search, include a study further strengthening the efficacy of DBT-A in reducing self-harm post-treatment, as well as a significantly lower number of suicide attempts and significantly fewer episodes of non-suicidal self-injurious behavior. Although no significant between-group differences were found at longer-term (12-month) follow-up in number of self-harm episodes, youths in the DBT-A were significantly more likely to show clinically significant change, defined as the absence of any self-harm, through the 12-month follow-up (52). Another study showed that young people with longer inpatient admissions were more likely to have multiple self-harm episodes, than young people treated with intensive community care (53).

Lastly, there were several unpublished protocol studies of RCTs for this subject population (54–58) and studies in progress (59–61), which we were unable to use for our systematic review. It is our hope that when these protocols are applied and published, such findings will greatly advance the field, and shed further light on effective treatments for adolescents at-risk for suicide.

Our updated systematic review suggests that given the heterogeneity of suicidal behavior, understanding which type of intervention is most effective for adolescents at risk of suicide can be a challenging but nonetheless paramount endeavor that requires further attention.

## Author contributions

UI and NS conducted the update to the systematic review, including the literature search, and analysis, UI and NS wrote the draft of the paper, JRA, PM, and TT added clinical and critical insight to the overall paper structure, and DO supervised the procedure and the overall study.

JRA has received grant, research, or other support from the National Institute of Mental Health, the American Foundation for Suicide Prevention, the Substance Abuse and Mental Health Services Administration, the American Psychological Association (APA), the Society of Clinical Child and Adolescent Psychology (Division 53 of the APA), and the Association for Child and Adolescent Mental Health. She has served as a consultant on quality improvement for depression and suicide/self-harm prevention, serves on the Scientific Council of the American Foundation for Suicide Prevention, and the Scientific Advisory Board of the Klingenstein Third Generation Foundation.

PM is a co-applicant to the following grant: Mars B, Gunnell D, Joinson C, Moran P, Relton C, Hemani G, Heron J, Suderman M, Ford T. Pathways to self-harm: Biological mechanisms and genetic contribution. MRC. £297,867. 2017-2019 Co-applicant (to PM).

### Conflict of interest statement

The authors declare that the research was conducted in the absence of any commercial or financial relationships that could be construed as a potential conflict of interest.
